# Report on Criminal Abortion

**Published:** 1868-04

**Authors:** J. C. Stone

**Affiliations:** Burlington, Iowa


### REPORT ON THE SUBJECT OF CRIMINAL ABORTION

BY J. C. STONE, M. D., OF BURLINGTON, IOWA.

ABORTION was practiced to an alarming extent among all the earlier nations, excepting only among the Jews. Aristotle and Plato defended it. It was denounced by the earlier Christians, but was common in Europe through the middle ages, and still prevails among the Mohammedans, Chinese, Japanese, Hindoos, and most of the nations of Africa and

Polynesia to such an extent, that it has been said, that "we may well doubt whether more have ever perished in those countries by plague, by famine, and the sword."

We need not go back to the middle ages or the time of the early Christians for examples of this crime. Here, in this country, in the middle of the nineteenth century, in our cities and towns where literature, science, morality and christianity are supposed to have great influence—where all the domestic and social virtues are reported as being in full and delightful exercise—even here in our own land, and in our own day, individuals, male and female, exist who are continually imbuing their hands and consciences in the blood of unborn infants! Yea, even *medical* men are to be found who are too lazy to work, too ignorant to think, and too heartless to feel, who, for a pecuniary recompense, will poison the fountains of life, or forcibly induce labor to the certain destruction of the fœtus, and not unfrequently to the destruction of the parent.

The moral sense of the community is so low on this subject that mothers, in many instances, in order to get rid of the fruits of illicit pleasure, do not hesitate to destroy their own progeny, in violation of every natural sentiment, and in opposition to the laws of God and man. Married women, also, from fear or dread of labor, more frequently from indisposition to have the trouble, the care, or the expense of children, or for some other motive equally trifling and degrading, solicit the destruction of the embryo by their medical attendant, and, when informed of the nature of such transactions, you get an expression of pretended or real surprise, that any one should deem the act improper; yet, in spite of the solemn warning of the physician, they double their guilt by making application to the debased and murderous charlatan who, for a piece of silver, will annihilate the life of a fœtus, and if not of the guilty mother — will have caused injuries that will result in untold physical suffering during the remainder of a miserable existence.

If this low estimate of fœtal life was confined to the ignorant, or to the lower classes of society, some allowance should be made; but such is not the case. The educated, refined, and so-called moral woman, who occupies a high station in society, whose influence in the social circle is greater, and whose character has not been suspected, will deliberately employ any and every means which may destroy her unborn offspring.

Unwelcome as the truth may be, it must be acknowledged that one of the fruits of advancing civilization is growing disregard of the claims of offspring upon families and individuals who should be their protectors. In one word, this crime of fœticide—this wilful and deliberate murder of unborn children—is being popularized! Criminal abortion is practiced and defended, not alone in the courts of kings, but in hamlets of their subjects; not exclusively in the great commercial centres, but in nearly every town and village throughout the country.

So general is the practice, so pernicious its influence upon community, and so terrible are the physiological consequences to its victims, that it is demanded of us, as conservators of the public health and morals, that we bring the weight of our professional characters to counteract and disprove the shallow sophistry upon which the practice is founded.

If we examine the statistics* upon this subject, we find that in the City of New York, from 1805 to 1849, the ratio of fœtal deaths to the whole population had increased from 1 in 1,663 to 1 in 340. Two years later in France, the ratio was only about 1 in 1,000.

The actual number of fœtal deaths in New York nearly doubled from 1850 to 1857! Compared with the total number of births, including illegitimate cases, the highest rate of fœtal mortality in foreign countries is that of Belgium. This is 1 in 16.8; but in this free Christian land, the proportion is 1 to 8. In New York, from 1805 to 1855, the number of fœtal deaths, as compared with the total mortality, had increased from 1 in 37 to 1 in 13. In 1846, the proportion of abortions to still-births, at full term, was about 1 in 10; ten years later, about 1 in 4.

The statistics of Massachusetts† and other States mark an exhibit equally frightful, and yet it is probable that these tables only approximate the truth; for, while nearly every case of still-birth and accidental abortion is recorded, it is a legitimate presumption that thousands of forced abortions, from the very nature and necessity of the case, never find a place upon the public register. But the sworn secrecy of parties engaged in this nefarious pursuit and practice can not suppress the inference that a terrible responsibility exists somewhere, and that medical men, knowing these facts, should exert themselves to put a stop to this worse than sacrifice of human life.

Nor is that all. Figures prove another fact that should startle, not only the medical mind, but thinking men of the entire country.

---

* Dr. Storer's Statistics of New York.

† Dr. Shurtliff's Statistics of Massachusetts.

Since 1850, the increase of births over deaths, in the State of Massachusetts, has been entirely of *foreign catholic origin.* But for the immigration of foreigners and their natural increase, the population of that great and wealthy State would not only not increase, but would actually decrease. When we consider, in addition to all this, that the ratio of children to each family has lessened by one-half! The conlusion is easy; for it cannot be said that the present generation of parents have suffered sexual or social changes that tend to make them less prolific than their ancestors. It follows, therefore, that the cause is not one of disability, but simply one of disinclination. It is the result of a great and growing crime—it is not passive or accidental, but premeditated and intentional.

What is true of Massachusetts and New York, is true in a greater or lesser degree of every State, and Iowa fills her quota of crime as surely as she filled the broken ranks of her regiments during the late war.

The great facilities for intercourse between distant cities and communities is fast making the whole country metropolitan. The fashions of the city, to-day, are the fashions of country to-morrow. The great pity is that, in the race of progress, crime outstrips virtue.

American ideas, manners and opinions, are in a great measure gathered from newspapers and periodicals teeming with police reports, criminal trials, and shocking developments that transpire daily. These are brought to the eye and mind by the press, are assimilated, correlated either to good or bad purposes and ends by the multitude. Nothing follows more naturally than a corresponding laxity of morals in matters the most sacred, and which should be kept from the public eye. Men uneasy in their married state measure the period of such relation by the railroad time-table from New York to some town in Indiana, where the moral obliquity of the court points to certain success, and divorce is obtained for a fee just a little above that fixed by law in other cases. The seductionist, (for it is reduced to a trade) troubled by the foreshadowing of the fruits of his illicit commerce, has but to apply to the professional abortionist, who is his friend, and will extinguish the vital spark with less of feeling and hesitation than one would have in pulling down a cobweb or plucking a flower.

These are facts too well known to every practitioner to require discussion; proof positive may be wanting, but evidence sufficient to convince the mind is but too frequently forced upon us in our everyday experience; and worst of all, go where we will, and we are brought into competition with individuals claiming to be *medical men*, crafty and designing, who do not hesitate to disgrace themselves, dishonor the profession to which they pretend to belong, who deliberately sell out their birthright to be numbered among the noble sons of our art for a miserable mess of pottage, by yielding to the importunities of those who insist that this abominable work of abortion shall be done at any cost, even at the risk of their own lives.

All must be more or less familiar with the stereotyped pleas for the induction of fœticide, against which we are urged by every consideration of rightful duty, by the laws of physiology, by an established code of morals, and by the political law of all well regulated communities. One plea set up is, that prior to quickning, the embryo is a mass of inanimate matter, not yet endowed with vitality! This argument I am sorry to say is still urged by some physicians, and is believed by thousands of the common people, and we hear it reiterated by those who should know better, I think sometimes simply in extenuation of their guilt.

Once the conditions for conception are supplied, and the vitalizing portion of the semen has impressed itself upon the ovum, somewhere along the course of the generative track, the first step in the reproductive series is taken. From this time forward, whatever imperils the integrity of that germ implicates life; and he who intentionally intercepts the wonderful changes incident thereto, is a veritable murderer—no more, no less. Whether prior, or subsequent to the formation of the placentæ, the dependence upon the mother must be substantially the same. It appears to me that no one in these advanced days of knowledge on this subject can doubt for a moment the physical laws that regulate supply and waste, the nutrition and detritus of germ life, embryonic life and fœtal life, are identical, and there is nothing in the mode of their operation that could possibly lead us to infer that the process of intra-uterine life is not from the moment of fecundation of the greatest importance.

The fertilized human ovum is not like the seed that has been wrapped in some old mummy, and left to await for ages the conditions for its development. Its growth is steady and progressive, physiological and positive. If the mass were inanimate or dead, it would be simply a foreign body, which the organs would reject and cast off long before the period of the so-called quickning. The very fact that it is not cast off is evidence of its life, and is so regarded in practice in cases of threatened abortion.

The danger to the mother in consequence of abortion occurring at any period of gestation are many and imminent, and are so many additional reasons against the crime of which we are speaking. The risk of death from hemorrhage, peritonitis, pelvic cellutitis, or phlebitis ; or she may succumb to the shock and drain upon her nervous and nutrative resources ; or if she escape the extreme direct consequences of such infractions upon physical law, still she is in great danger lest the sudden and forcible interruption of the textural changes and sympathetic relations peculiar to pregnancy, result in more or less of disease and disorder. The ovaries, the mammary glands, the uterine walls, vessels and lining membrane, and the nutritive and nervous system are especially likely to suffer; and, strange to say, the earlier the period of abortion the greater the danger and liability to indirect consequences. The list is a long one. It includes the different forms of ovarian inflammation, ovarian dropsy, every species of menstrual derangement, chronic and sub-acute inflammation of the womb, metro-peritonitis, moles, uterine polypi, hydatids and fibroids, uterine displacements, uterine and vaginal fistulæ, subsequent abortion, atresia of the cervix uteri, sterility, hysteria, dyspepsia, neuralgia, leucorrhœa, tetanus, and mania.

With this array of the more than possible physical consequences of fœticide, we pass to the consideration of the moral reason against the crime, with the feeling that there is nothing in the study of physiology or pathology but what condemns the practice.

Poverty is one of the most frequent arguments adduced in favor of fœticide. Another party insist that no more children can be born with safety to the mother; while others plead ill-health or indisposition. Many dread the responsibility which would be entailed upon them ; and some there are who are not willing to sacrifice their youthful appearance, blooming looks, and the graceful, girlish manners, that otherwise they vainly hope to perpetuate. It would be a very loose code of morals that recognized the validity of either of these arguments. Who does not know that, but for the providence that blessed them with children in their early married life, many parents would be houseless, homeless, and friendless in their old age? How many sons are the prop and stay of fathers in this and every other country? How many daughters are proud of the mothers that have been spared them, and whose motherly love, sympathy and counsel they secure by giving a place in a home that would be desolate without them? Why, children are better investments than money in government bonds; for it secures a competency and position, comfort and happiness. The mutability of fortune may deprive them of house and lands, and all the means for self-support be snatched away, but these may not be sacrificed.

Nor can we justify the plea that is based upon the mother's ill-health. If it were not a physiological rule, with but few exceptions in point of fact, that the course and progress of most diseases are held in check by pregnancy, it would still be much better that the woman bear the ills she has than for her to fly to others of which she is ignorant.

Young and beautiful wife, what are your charms, that you are ready to sacrifice all that is true, and noble, and exalted in character to them? You may look in vain for the elixir of life; not one of the many inventions and expedients of the toilet so adds to the personal beauty and attractiveness, or so surely perpetuates loveliness of feature, and all that is delightful and admirable in tone and figure, in grace and symmetry, as the reflex of the nursery, and the smiles and cheer that well up, and spring with unceasing freshness and newness from the hearts of children. God has ordained but one mode of perpetuating loveliness of feature; that mode is entirely consonant with the most exalted character. No sight is so beautiful, so suggestive, so full of character, as of a mother surrounded by her children—no picture half so lovely. What a vandal is he who would destroy it, and who, under the flimsy pretense of preserving a butterfly, would murder a child, and ruin a woman.

What must we do? A public opinion should be created upon the subject reflecting the truth, without fear or favor, upon the crime.

Even if legislation were practicable in any case, it can do little until this custom is branded *as* a crime; until the unwarranted discrimination between the murder of a child in one condition of its being, and that in another, is broken down, and a social judgment shall condemn each equally as a violation of human and natural law, and until the opinion everywhere prevails that the due course of maternity is not a disease to be treated, but a course of nature which cannot be interrupted without calamitous results.

Nothing but moral influences of the most persistent kind can diminish the number of American women who, for selfish and personal ends, butcher or poison their children, while looking with holy horror upon devotional mothers of India who, in the conscientious discharge of their religious duties, throw their babes into the Ganges.

